# A randomized controlled trial to evaluate the effectiveness of different methods on pain management during orthodontic debonding

**DOI:** 10.1186/s40510-022-00401-y

**Published:** 2022-03-01

**Authors:** Sanjay Prasad Gupta, Shristi Rauniyar, Pravin Prasad, Pranil Man Singh Pradhan

**Affiliations:** 1 Department of Orthodontics and Dentofacial Orthopedics, Tribhuvan University Dental Teaching Hospital, Maharajgunj Medical Campus, Institute of Medicine, Tribhuvan University, Maharajgunj, Kathmandu, Nepal; 2 Dental Villa-Orthodontic Center & Speciality Dental Clinic, Balkhu, Kathmandu, Nepal; 3 Department of Clinical Pharmacology, Maharajgunj Medical Campus, Institute of Medicine, Tribhuvan University, Maharajgunj, Kathmandu, Nepal; 4 Department of Community Medicine, Maharajgunj Medical Campus, Institute of Medicine, Tribhuvan University, Maharajgunj, Kathmandu, Nepal

**Keywords:** Debonding, Finger pressure, Medication, Pain management, Stress relief

## Abstract

**Background:**

Orthodontic treatment procedures like separator placement, archwire placement, orthodontic force application, miniscrew placement and debonding procedure usually involve pain and discomfort. Pain perception and methods to reduce pain during debonding in regard to gender and different locations of oral cavity is still a poorly documented issue in orthodontics. The aim of this study was to evaluate the effectiveness of different methods on pain management during debonding and its association with gender and location.

**Materials and methods:**

One hundred and forty orthodontic patients in the stage of debonding were randomly assigned into four groups according to different methods used during debonding; Group A: Medication group (Paracetamol given 1 h before debonding), Group B: Finger pressure group, Group C: Stress relief group and Group D: Control group. A visual analog scale (VAS) was used to assess the pain intensity just after debonding for each sextant.

**Results:**

Among 140 participants, 61 (43.57%) were males and 79 (56.43%) were females. Differences in VAS score in different areas of oral cavity among all groups were found to be significant (*p* < 0.05). Total VAS score was greater in control group (16.67) followed by stress relief group (13.33) and finger pressure group (10) and least in medication group (8.33). The VAS score was higher in the upper front and lower front sextants in all the groups. Females reported higher VAS score and in upper front sextant, it showed significant difference (*p* = 0.018). On comparison, total VAS scores were statistically significant difference in medication-stress relief arm pair (*p* = 0.009), medication-control arm pair (*p* < 0.001) and finger pressure-control arm pair (0.002). The total VAS score comparison between medication-finger pressure arm was not significant (*p* = 0.172).

**Conclusions:**

Pain perceived during debonding varies in different areas of oral cavity among all the groups. Anterior area of oral cavity and female seems to be more sensitive to pain. Use of finger pressure can be used effectively for pain management during debonding.

## Background

Orthodontic treatment procedures like separator placement, archwire placement, orthodontic force application, miniscrew placement and debonding procedure usually involve pain and discomfort and up to 95% of patients experienced pain during orthodontic treatment [1–3].

Pain is a subjective response which shows large individual variations. Pain perception may be related to various factors such as age, gender, individual pain threshold, motivation, cultural differences, psychological condition, previous negative dental experience, and the magnitude of orthodontic force. Pain could be somatization of either anxiety or depression so the pain perception may be influenced by the anxiety of the person [2]. Some studies showed that female reported more pain experience than male [4, 5], while other studies showed no gender differences regarding pain perception [6, 7].

Debonding procedure should be harmless, painless and quick [8]. The pain during debonding can be minimized by various means like the use of different orthodontic instruments, laser application, analgesics, ultrasound, adjunctive procedures, thermal heating the orthodontic adhesives, or biting occlusal bite wafers at debonding [7, 9–11].

Study by Williams and Bishara suggested that pain while debonding can be managed by applying finger pressure or by asking the patient to bite on a piece of cotton roll while debonding [12] while other study described the use of an occlusal rim wax for pain-free debonding [13].

The stress relief method was also tried to reduce the pain during bonding which is based on cognitive behavior therapy and mainly directed against the psychological mechanism of pain in the patients [14].

Previous studies fail to compare different pain management methods during debonding in regard to gender and on different locations of oral cavity using randomization, placebo and blinding [7, 9–12].

Along with this, recent systematic review revealed that there is weak evidence on different pain management methods like use of finger pressure and analgesics on pain perception during orthodontic debonding [15]. Hence, we have conducted this trial to address the previous issues in a robust methodological process.

As a clinician, our aim is to provide painless debonding to the patients as much as possible so the objective of this study was to evaluate the effectiveness of different methods on pain management during orthodontic debonding along with its association with gender and location.

## Methods

This study was approved by Ethical Review Board of Nepal Health Research Council (Ref. 139/2020) and was registered in Clinical trials Registry-India (CTRI/2020/08/027272) before commencement of the study. This study was conducted according to the CONSORT 2010 guidelines [16]. Written consent was obtained from the patients prior to participation.

The mean difference of 15 mm was proved to be clinically significant impact on a visual analog scale (VAS) [17]. Standard deviation of 19.6 was calculated from a previous study [18]. A power analysis revealed for maintaining a ᾳ = 0.05 and a power of 80%, a sample of 27 subjects per group was required. To compensate for potential dropouts, 35 patients were enrolled in each group.

The patients aged between 13 and 30 years who could understand, assess and answer the questionnaires**,** undergoing treatment of both arches with 0.022 MBT double mesh base metal bracket and 0.019 × 0.025 inch stainless steel finishing arch wires present for at least two months were included in this study.

Exclusion criteria were patients with history of taking medicine periodically or in the last 24 h (e.g. analgesic, anti-inflammatory and anxiolytic), Generalized Anxiety Disorder 7-item scale (GAD-7) score of ˃8, debonded brackets at the time of debonding, missing teeth except extracted premolars, active periodontal problems (recession and mobility greater than Grade I), heavily restored or root canal treated tooth, craniofacial deformities that would effect dentoalveolar bone quality (e.g. cleft lip and palate), surgical treatment (including impacted tooth removal) and presence of miniscrews.

### Allocation, concealment and randomization

A random sequence was generated using a computer random number generator (Stat Trek programme; https://stattrek.com/). One hundred and forty opaque envelopes (35 for each intervention) were prepared and kept inside a bowl. Envelopes were coded and sealed by another investigator who was not involved in selecting the patients for debonding.

Patients were assessed for eligibility and one hundred and forty orthodontic patients in the stage of orthodontic debonding from Department of Orthodontics and Dentofacial Orthopedics of Tribhuvan University Dental Teaching Hospital & Dental villa-Orthodontic Center & Speciality Dental Clinic, Kathmandu, were selected from August 21, 2020, to May 31, 2021.

**Fig. 1:**
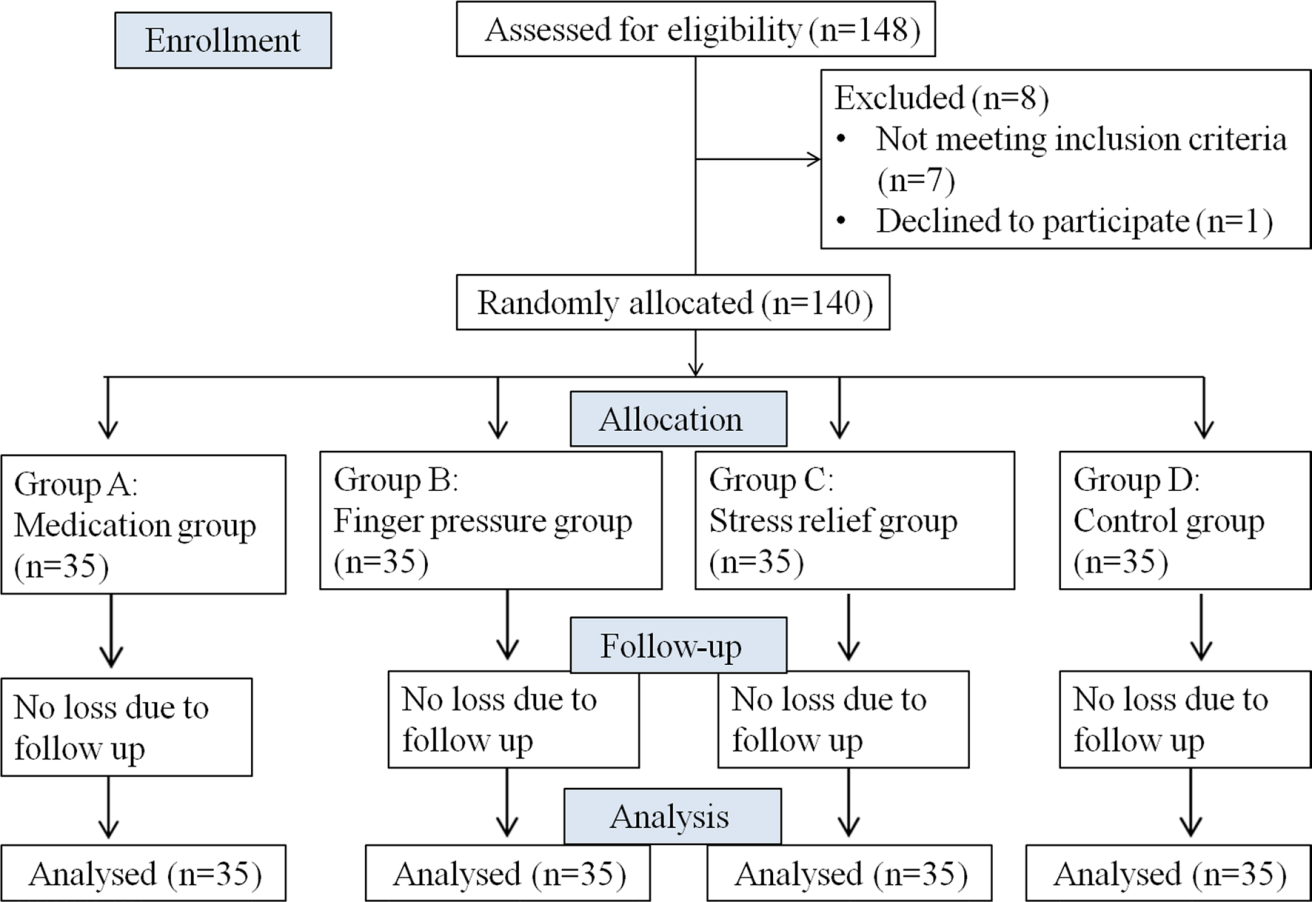
CONSORT flow diagram

Each patient was asked to select one envelope and accordingly each patient was assigned the intervention based on his or her envelope (Fig. 1).

Patient was randomized into four debonding groups:

- Group A: Medication group-A single dose of 500 mg Paracetamol tablet (NIKO, National pharmaceuticals, Nepal) was given 1 hour before debonding.
- Group B: Finger pressure group-During debonding of each bracket, operator’s finger pressure was applied from the occlusal surface of the tooth in a gingival direction with the thumb. A cotton pad was held under the thumb to eliminate the effect of occlusal morphological variations.
- Group C: Stress relief group-Patients were instructed to open their mouths and not to occlude. To relieve their stress, instructions were given that debonding would not cause harm or serious pain. This method is based on cognitive behavior therapy which is based on psychological mechanism of pain in patients [14].
- Group D: Control group-Patients were instructed to open their mouths and not to occlude. Routine debonding procedures were followed without any advice or application of any methods.

### Blinding

The patients were blinded and did not know which group he or she belongs to by giving placebo tablets (having similar packaging as that of paracetamol tablet) to all groups except medication (Paracetamol) group. The treating orthodontist was also partially blinded between medication (group A) and control group (group D) as he did not know which envelope contained paracetamol tablet and which contained placebo. However, due to the nature of the treatment, treating orthodontist knew if the patient was assigned to finger pressure group (group B) or stress relief group (group C).

**Fig. 2:**
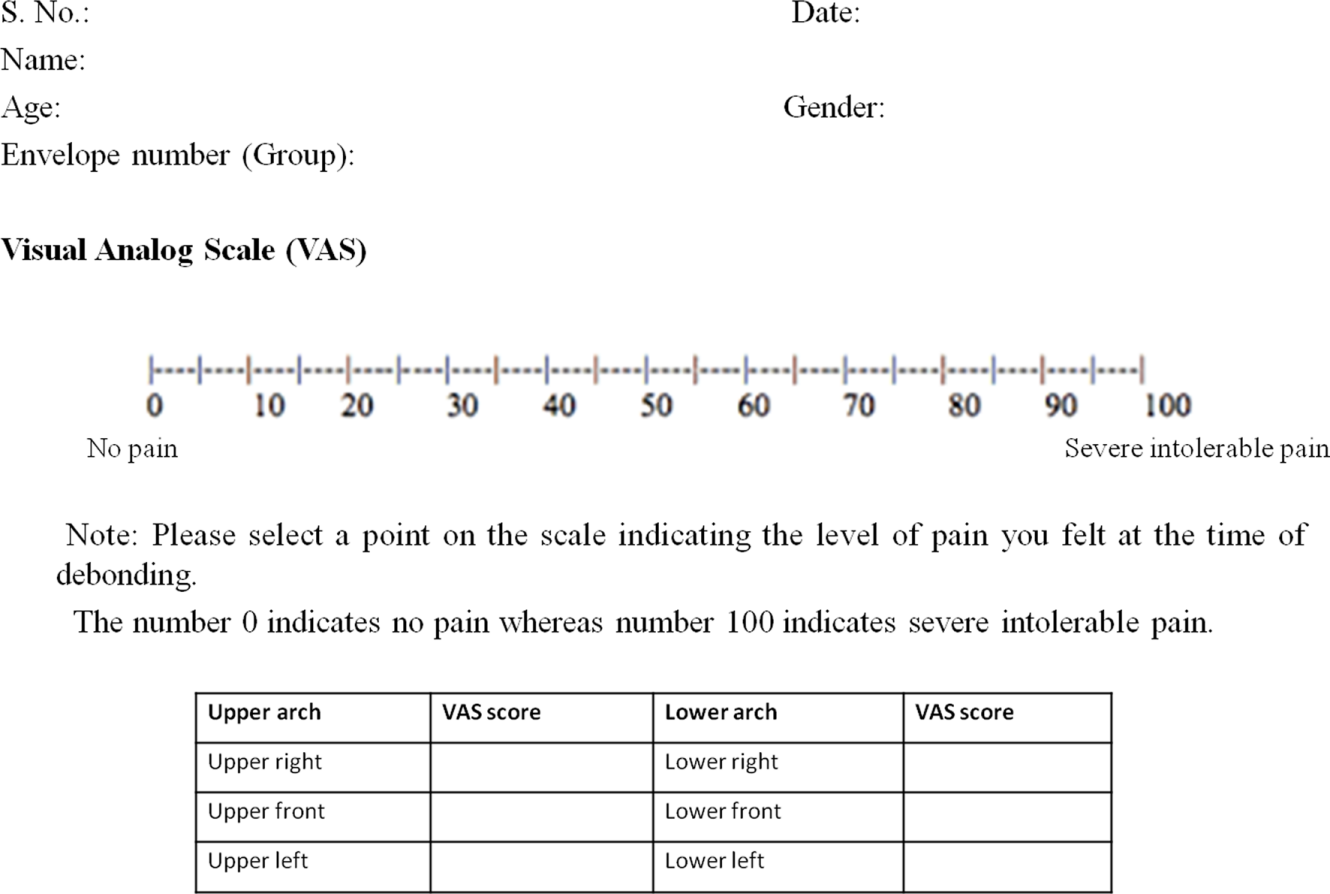
Pro forma of the study used to collect data using the visual analog scale (VAS)

The patient’s levels of anxiety and fear of pain were evaluated at the time of enrollment by GAD-7 score [19] and anxiety score of ≤ 8 was selected. The score of above 8 is considered as the moderate to severe levels of anxiety which might influence the VAS score; hence, those participants were excluded from this study. A 100-mm VAS was used to assess pain intensity just after debonding. This scale was composed of a millimeter ruler; the number 0 indicates no pain whereas number 100 indicates severe intolerable pain (Fig. 2). Before debonding, each patient was instructed about the study objectives and explained that at the end of debonding, it would be necessary to assess the pain intensity of the procedure using a VAS in each sextant; Upper right (UR), Upper front (UF), Upper left (UL), Lower right (LR), Lower front (LF) and Lower left (LL).

All the debondings were performed by the same clinician (SPG) with the same bracket removal plier (Eltee, Libral traders, India), starting from the upper and lower right sides of the jaws, respectively. The archwires were in situ during debonding.

Data obtained were coded and transferred to MS-excel sheet. The data were verified and analyzed statistically using SPSS Statistics Version 21.0 (Armonk, NY: IBM Corp.) with confidence level set at 95% (*p* < 0.05) to test for significance. Data were descriptively analyzed. Normality of the data was tested using Kolmogorov–Smirnov and showed non-normally distributed. Kruskal–Wallis was applied to find out any statistically significant difference in the VAS pain score in different areas of oral cavity among the groups and for intergroup comparisons. The Mann–Whitney test was used for comparing VAS score between males and females. Categorical data analyses were carried out by the Chi-square test. The VAS scores were presented in median along with minimum and maximum values.

## Results

A CONSORT diagram showing the flow of patients through the study is depicted in Fig. 1. There were 148 participants assessed and approached for the study, of which 140 participants were selected and completed the study. The gender-wise and age distribution of the participants according to the group/treatment arm has been summarized in Table 1. Gender-wise comparison was not found to be significant.

**Table 1:** Gender-wise and age distribution of the participants according to the treatment arms compared using Pearson Chi-square test

Variables	Groups/treatment arms				All groups	*p* value
	Medication	Finger pressure	Stress relief	Control		
*Gender*						
Male	15 (42.9%)	12 (34.3%)	20 (57.1%)	14 (40%)	61 (43.57%	0.257
Female	20 (57.1%)	23 (65.7%)	15 (42.9%)	21 (60%)	79 (56.43%)	
Age (years; mean ± SD)	21.74 ± 4.8	18.40 ± 4.5	21.83 ± 3.6	22.31 ± 6.5	21.07 ± 5.1	0.004*

*p < 0.05 = Statistically significant

It was seen that higher pain score was reported in control arm in most of the sextants by the participants. The difference in VAS score was compared using the Kruskal–Wallis test and was found to be significant in all areas of oral cavity among all groups (*p* < 0.05). It is summarized in Table 2.

**Table 2:** Visual analog scale (VAS) score according to the treatment arms compared using Kruskal–Wallis test

Areas of oral cavity	Groups/treatment arms Median VAS score (minimum, maximum)				*p* value
	Medication	Finger pressure	Stress relief	Control	
Upper right	5 (0,30)	5 (0,30)	10 (0,25)	10 (0,40)	0.029*
Upper front	10 (0, 40)	15 (0, 40)	15 (5, 50)	25 (5, 60)	0.001*
Upper left	5 (0, 30)	5 (0, 20)	10 (0, 20)	10 (0, 35)	0.018*
Upper total	6.67 (0, 30)	8.33 (0, 26.67)	10 (1.67, 30)	13.33 (1.67, 36.67)	0.001*
Lower right	5 (0, 40)	10 (0, 30)	10 (0, 20)	10 (0, 60)	0.014*
Lower front	20 (0, 30)	20 (5, 40)	20 (10, 50)	30 (10, 70)	0.00008*
Lower left	5 (0, 30)	5 (0, 35)	10 (0, 40)	10 (0, 30)	0.010*
Lower total	10 (0, 33.33)	13.33 (1.67, 30)	13.33 (3.33, 30)	16.67 (3.33, 43.33)	0.0001*
Total	8.33 (0.83, 26.67)	10 (0.83, 25)	13.33 (2.50, 30)	16.67 (3.33, 43.33)	0.0001*

*p < 0.05 = Statistically significant

The median value of total VAS score was highest in control group (16.67) followed by stress relief group (13.33) and finger pressure group (10) and least in medication group (8.33). The median values of VAS score were higher in the upper front and lower front sextants irrespective of the groups.

Females were found to report higher median score with upper front area showed significant difference (*p* = 0.018) as shown in Table 3.

**Table 3:** Comparison of VAS score in different areas of oral cavity by gender using Mann–Whitney test

Areas of oral cavity	Median VAS score (minimum, maximum)		*p* value
	Male	Female	
Upper right	5 (0,25)	10 (0, 40)	0.185
Upper front	10 (0, 50)	20 (0, 60)	0.018*
Upper left	5 (0, 20)	10 (0, 35)	0.362
Upper Total	6.67 (0,30)	11.67 (0,36.67)	0.06
Lower right	10 (0, 30)	10 (0, 60)	0.542
Lower front	20 (5, 50)	20 (0, 70)	0.474
Lower left	10 (0, 40)	10 (0, 30)	0.467
Lower Total	13.33 (1.67, 30)	13.33 (0, 50)	0.767
Total	10 (0.83, 30)	13.33 (0.83, 43.33)	0.241

*p < 0.05 = Statistically significant

**Fig. 3:**
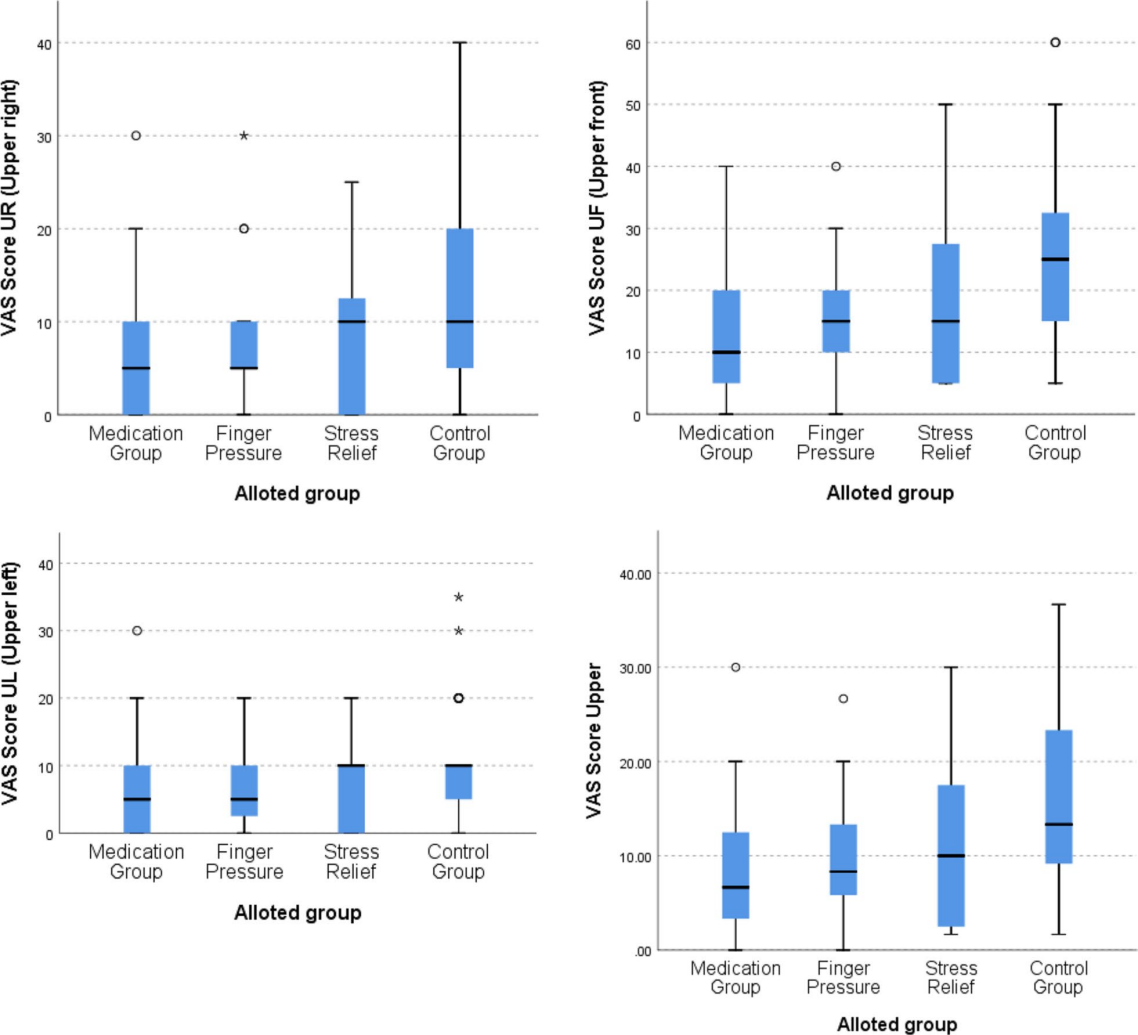
Boxplot graph comparing VAS score in four treatment arms/groups in upper right (top left), upper front (top right), upper left (bottom left) and upper jaws (bottom right)

On pairwise comparison for maxilla, it was seen that medication group had lower VAS score than control group in all sextants and as maxilla total. The differences were found to be significant as depicted in Fig. 3 and Table 4.

**Table 4:** Pairwise comparison among different treatment arms of VAS score in different areas of upper and lower jaw using independent Kruskal–Wallis test

Group pair	*p* value								
	UR	UF	UL	UT	LR	LF	LL	LT	T
Medication—finger pressure	0.136	0.526	0.242	0.327	0.416	0.013*	0.748	0.077	0.172
Medication—stress relief	0.076	0.214	0.066	0.09	0.019*	0.002*	0.036*	0.002*	0.009*
Medication—control	0.003*	0.0001*	0.002*	0.0001*	0.004*	0.00001*	0.004*	0.00002*	0.00001*
Finger pressure—stress relief	0.778	0.544	0.504	0.474	0.128	0.505	0.076	0.169	0.215
Finger pressure—control	0.136	0.002*	0.055	0.005*	0.039*	0.041*	0.011*	0.014*	0.002*
Stress relief—control	0.227	0.012*	0.210	0.035*	0.590	0.168	0.450	0.285	0.073

*UR* upper right, *UF* upper front, *UL* upper left, *UT* upper total, *LR* lower right, *LF* lower front, *LL* lower left, *LT* lower total, *T* total of upper and lower

*p < 0.05 = statistically significant

**Fig. 4:**
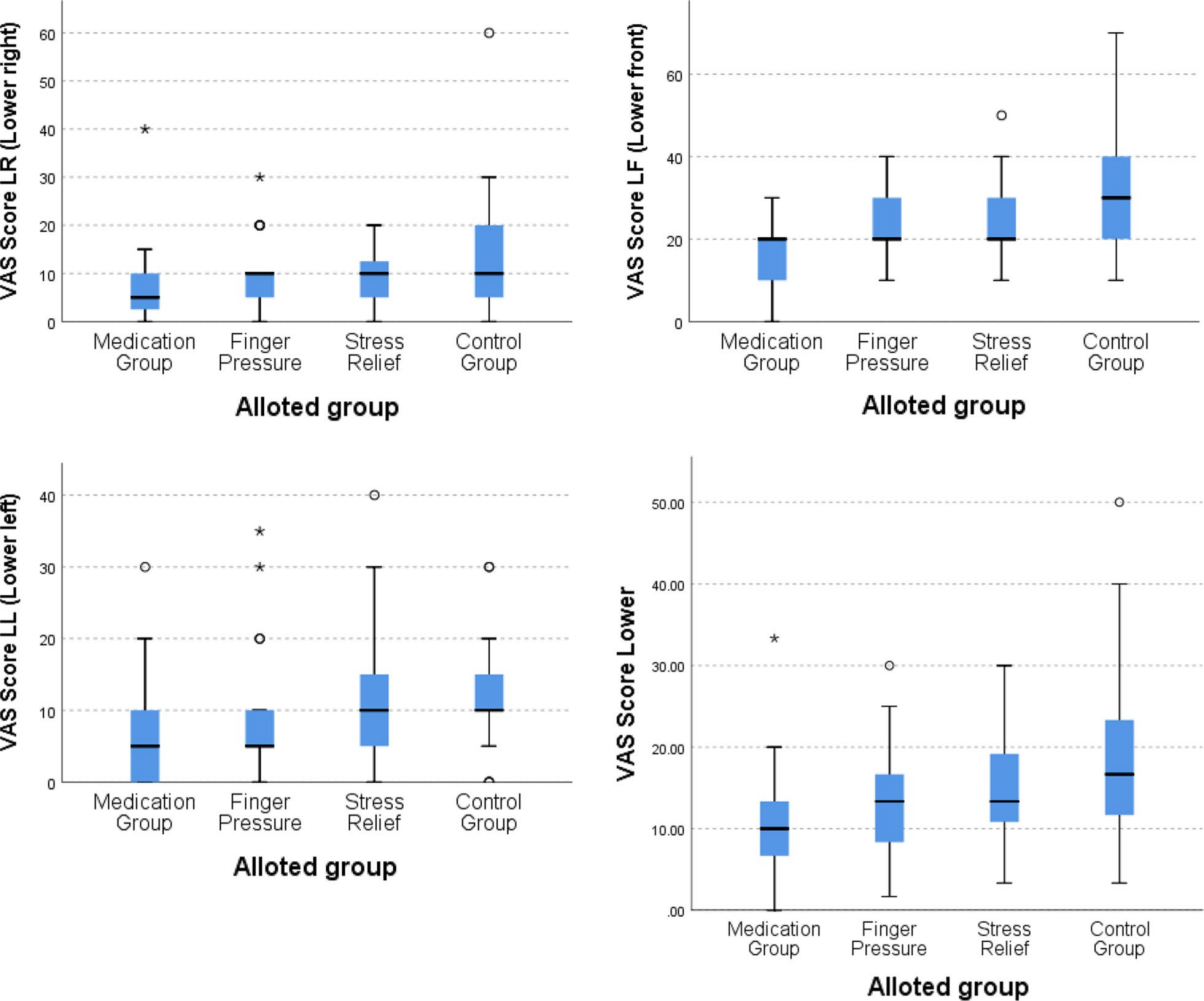
Boxplot graph comparing VAS score in four treatment arms/groups in lower right (top left), lower front (top right), lower left (bottom left) and lower jaws (bottom right)

Differences in VAS score were also seen in sextants of mandible as depicted in Fig. 4 and Table 4.

**Fig. 5:**
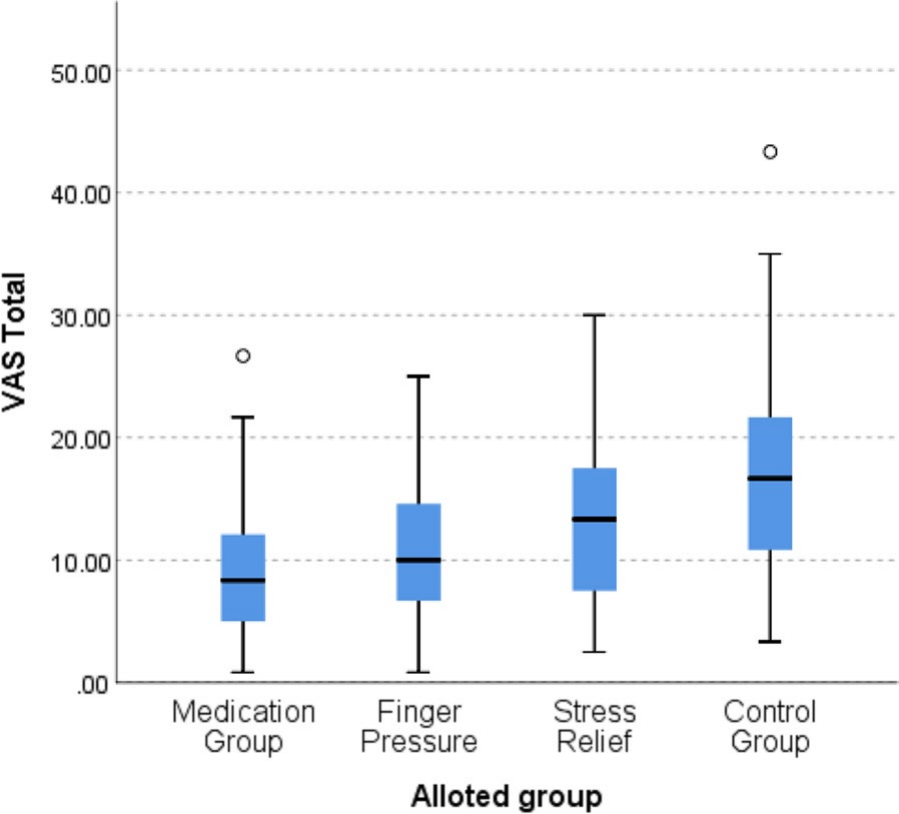
Boxplot graph comparing total VAS score in four treatment arms/groups in all areas of oral cavity

When total VAS score of all sextants was added and pairwise comparison was done, significant differences were observed in medication-stress relief arm pair (*p* = 0.009), medication-control arm pair (*p* = 0.00001) and finger pressure-control arm pair (*p* = 0.002) but no significant difference between medication-finger pressure arm pair (*p* = 0.172) as shown in Table 4 and Fig. 5.

## Discussion

This clinical trial was designed to evaluate the effectiveness of different methods on pain management during debonding. The effects of other important determinants like gender as well as different location on pain were also observed.

To quantify the pain intensity of the patient, various scales are commonly used like visual analog scale (VAS), numerical rating scale and verbal rating scale (VRS). Comparative studies regarding these scales showed no statistically significant difference among them [20, 21]. In this study, VAS was used because of its ease of application.

There are many methods used for debonding the bracket. Khan et al. [22] compared the ultrasonic/sonic instruments with the debonding plier and concluded that although sonic/ultrasonic based debonding technique is less painful approach but it is not recommended as a routine method as it is the most time-consuming approaches compared to debonding plier. In this study, all the patients were debonded with the same bracket removing plier to standardize the procedure. We choose bracket removing plier as the debonding instrument as it is inexpensive and most widely used in our part of the world.

Pain could be somatization of either anxiety or depression. Patient’s levels of anxiety and fear of pain were assessed at the time of participants enrollment which might affect the pain perception during debonding, so the patient who had anxiety score of ≤ 8 (evaluated by GAD-7 scoring) [19] was selected in this study to control the bias that is VAS score was less influenced by the anxiety of the participants. To control the inter-operator bias, single operator (SPG) performed all the debonding procedure with the same designated technique.

Various pain-relieving methods have tried to minimize the pain during orthodontic debonding. We advised the patient to take 500 mg of paracetamol one hour before the debonding in the medication group to control pain.

Evidence suggested that paracetamol is an effective and safe choice for the orthodontic pain at the usual therapeutic doses of 325 to 1000 mg/dose (10–15 mg/kg/dose in children) [23–25]. Kaur et al. tried 500 mg of paracetamol to control the separator pain and found it as effective method [26], whereas study by Priya et al. [10] concluded that use of analgesic (Paracetamol and Ibuprofen) 1 h before the debonding reduces the pain perception.

We have chosen the finger pressure method and stress relief method as it is easy, inexpensive and does not need extra armamentarium. Comparison of these methods was made with the medication and control group as well.

The results of this study revealed that medication group had least total VAS score (8.33) in comparison with finger pressure group (10), stress relief group (13.33) and control group (16.67), indicating that medication (Paracetamol) seems a better method of pain management than finger pressure and stress relief method when comparing it with the control.

There were statistically significant differences in the VAS score in different areas of oral cavity among all the groups. The median values of VAS score were highest in the lower front quadrant followed by upper front quadrant whereas least in upper and lower posterior region in all the groups, suggesting that anterior region of jaw is more sensitive to pain while debonding. The similar finding was reported by other studies [10, 11, 27–30]. It might be due to their anatomic location and root morphology. Teeth residing in the upper and lower front sextants has got single root having less surface area and housing in a thinner cortical boundary and has to bear more force than the posterior sextants that has got multirooted teeth housing in a thicker cortical boundary [11]. Debonding force per unit surface area of the root is explained by tactile sensory threshold which is 1 g in the anterior region while it is around 5 to 10 g in the posterior region of the dental arch [15].

In this study, the total VAS score for finger pressure group was 10 and for stress relief group was 13.33 which was slightly higher than the study of Karobari et al. [27] that is VAS score of 6.59 for finger pressure group and 7.49 for stress relief group. Similarly, total VAS score of this study was also higher than the study by Bavbek et al. [14] which showed VAS score of 7 for finger pressure group and 9.1 for stress relief group. It might be due to different cultural background.

In gender-wise comparison, female recorded higher VAS score and on upper front sextant, it showed significant difference. These findings are in agreement with the results of the previous studies about the impact of gender on pain perception [1, 2, 10, 14, 28–31]. In contradictory to this, other study showed no gender differences in pain perception [7].

On intergroup comparison, there were significant differences in total VAS score between medication-control group, between finger pressure-control group and medication-stress relief group while it showed no significant difference when comparing it between medication-finger pressure group, between finger pressure-stress relief group and between stress relief-control group. It suggested that finger pressure can be the equally reliable method to medication regarding pain management during debonding. In addition to this, the finger pressure method seems to be more effective than the stress relief method on the basis of total VAS score between these two groups which is in agreement with the study by Bavbek et al. who conducted the study on efficacy of different methods to reduce pain during debonding of orthodontic brackets [14]. Finger pressure can be considered as an easy and effective technique of pain control, since it is inexpensive, less time-consuming, and less technique sensitive. Finger pressure method works as it applies intrusive force on the incisal or occlusal surface of the tooth which stabilizes the tooth and counteracts the torsional and sheer/peel debonding forces applied to the periodontal ligament during debonding. Along with this, it provides proprioceptive stimulus and is believed to reduce the pain according to the gate control theory [12, 28].

The stress relief method is based on cognitive behavior therapy which is primarily directed against the psychological mechanism of pain in patients [14]. Study by Koyama et al. also noted that positive expectations result in reduced pain experience and works by altering the brain mechanism [32]. It is known that patients who trust their doctors are more comfortable during orthodontic procedure. The stress relief method along with the finger pressure method can also be tried clinically and will be the scope of further study to control the pain and discomfort during debonding as it is easy, inexpensive and does not need extra armamentarium.

## Limitations

Limitation of this study is that this trial did not assess the diurnal variation (circadian rhythm) on the level of post-debonding pain as it may have a confounding effect. It has reported that the intensity of orthodontic pain fluctuates over the course of a day with the lowest pain intensity during the mid-day hours [15, 33, 34]. This could be the scope for future study. Apart from this, pain is considered as a result of complex phenomena having subjective nature and shows large individual variations. Pain perception may be affected by the individual pain threshold. So, these could be the scope for the further research.

## Conclusion

- Pain VAS score in different areas of oral cavity was found to be statistically significant difference among all the groups.
- Anterior area of oral cavity and female seems to be more sensitive to pain while debonding.
- Although medication group had lower VAS score in comparison with control group, the use of finger pressure can also be used as an effective method for pain management during orthodontic debonding as it is easy, inexpensive, less technique sensitive and less time-consuming.

## Data Availability

The datasets used and/or analyzed during the current study are available from the corresponding author on reasonable request.
